# New Mechanistic Insights, Novel Treatment Paradigms, and Clinical Progress in Cerebrovascular Diseases

**DOI:** 10.3389/fnagi.2021.623751

**Published:** 2021-01-28

**Authors:** Johannes Boltze, Jaroslaw A. Aronowski, Jerome Badaut, Marion S. Buckwalter, Mateo Caleo, Michael Chopp, Kunjan R. Dave, Nadine Didwischus, Rick M. Dijkhuizen, Thorsten R. Doeppner, Jens P. Dreier, Karim Fouad, Mathias Gelderblom, Karen Gertz, Dominika Golubczyk, Barbara A. Gregson, Edith Hamel, Daniel F. Hanley, Wolfgang Härtig, Friedhelm C. Hummel, Maulana Ikhsan, Miroslaw Janowski, Jukka Jolkkonen, Saravanan S. Karuppagounder, Richard F. Keep, Inga K. Koerte, Zaal Kokaia, Peiying Li, Fudong Liu, Ignacio Lizasoain, Peter Ludewig, Gerlinde A. S. Metz, Axel Montagne, Andre Obenaus, Alex Palumbo, Monica Pearl, Miguel Perez-Pinzon, Anna M. Planas, Nikolaus Plesnila, Ami P. Raval, Maria A. Rueger, Lauren H. Sansing, Farida Sohrabji, Charlotte J. Stagg, R. Anne Stetler, Ann M. Stowe, Dandan Sun, Akihiko Taguchi, Mickael Tanter, Sabine U. Vay, Raghu Vemuganti, Denis Vivien, Piotr Walczak, Jian Wang, Ye Xiong, Marietta Zille

**Affiliations:** ^1^School of Life Sciences, University of Warwick, Warwick, United Kingdom; ^2^Institute for Stroke and Cerebrovascular Diseases, McGovern Medical School, The University of Texas Health Science Center at Houston, Houston, TX, United States; ^3^NRS UMR 5287, INCIA, Brain Molecular Imaging Team, University of Bordeaux, Bordeaux cedex, France; ^4^Departments of Neurology and Neurological Sciences, and Neurosurgery, Wu Tsai Neurosciences Institute, Stanford School of Medicine, Stanford, CA, United States; ^5^Neuroscience Institute, National Research Council, Pisa, Italy; ^6^Department of Biomedical Sciences, University of Padua, Padua, Italy; ^7^Department of Neurology, Henry Ford Hospital, Detroit, MI, United States; ^8^Department of Physics, Oakland University, Rochester, MI, United States; ^9^Peritz Scheinberg Cerebral Vascular Disease Research Laboratory, Department of Neurology, University of Miami Miller School of Medicine, Miami, FL, United States; ^10^Biomedical MR Imaging and Spectroscopy Group, Center for Image Sciences, University Medical Center Utrecht and Utrecht University, Utrecht, Netherlands; ^11^Department of Neurology, University Medical Center Göttingen, Göttingen, Germany; ^12^Department of Neurology, Center for Stroke Research Berlin, Charité—Universitätsmedizin Berlin, Berlin, Germany; ^13^Department of Experimental Neurology, Charité—Universitätsmedizin Berlin, Berlin, Germany; ^14^Bernstein Center for Computational Neuroscience Berlin, Berlin, Germany; ^15^Einstein Center for Neurosciences Berlin, Berlin, Germany; ^16^Faculty of Rehabilitation Medicine and Institute for Neuroscience and Mental Health, University of Alberta, Edmonton, AB, Canada; ^17^Department of Neurology, University Medical Center Hamburg-Eppendorf, Hamburg, Germany; ^18^Berlin Institute of Health, Berlin, Germany; ^19^Department of Neurosurgery, School of Medicine, University of Warmia and Mazury, Olsztyn, Poland; ^20^Neurosurgical Trials Group, Institute of Neuroscience, The University of Newcastle upon Tyne, Newcastle upon Tyne, United Kingdom; ^21^Laboratory of Cerebrovascular Research, Montreal Neurological Institute, McGill University, Montreal, QC, Canada; ^22^Division of Brain Injury Outcomes, Johns Hopkins University, Baltimore, MD, United States; ^23^Paul Flechsig Institute of Brain Research, University of Leipzig, Leipzig, Germany; ^24^Clinical Neuroengineering, Center for Neuroprosthetics and Brain Mind Institute, Swiss Federal Institute of Technology Valais, Clinique Romande de Réadaptation, Sion, Switzerland; ^25^Clinical Neuroscience, University of Geneva Medical School, Geneva, Switzerland; ^26^Institute for Experimental and Clinical Pharmacology and Toxicology, University of Lübeck, Lübeck, Germany; ^27^Fraunhofer Research Institution for Marine Biotechnology and Cell Technology, Lübeck, Germany; ^28^Institute for Medical and Marine Biotechnology, University of Lübeck, Lübeck, Germany; ^29^Department of Diagnostic Radiology and Nuclear Medicine, University of Maryland, Baltimore, MD, United States; ^30^Department of Neurology, A.I. Virtanen Institute for Molecular Sciences, University of Eastern Finland, Kuopio, Finland; ^31^Burke Neurological Institute, White Plains, NY, United States; ^32^Feil Family Brain and Mind Research Institute, Weill Cornell Medicine, New York, NY, United States; ^33^Department of Neurosurgery, University of Michigan, Ann Arbor, MI, United States; ^34^Psychiatric Neuroimaging Laboratory, Brigham and Women’s Hospital and Harvard Medical School, Boston, MA, United States; ^35^Department of Child and Adolescent Psychiatry, Psychosomatic, and Psychotherapy, Ludwig Maximilians University, Munich, Germany; ^36^Lund Stem Cell Center, Lund University, Lund, Sweden; ^37^Department of Anesthesiology, Renji Hospital, School of Medicine, Shanghai Jiaotong University, Shanghai, China; ^38^Department of Neurology, The University of Texas Health Science Center at Houston, McGovern Medical School, Houston, TX, United States; ^39^Unidad de Investigación Neurovascular, Departamento Farmacología y Toxicología, Facultad de Medicina, Instituto Universitario de Investigación en Neuroquímica, Universidad Complutense de Madrid, Madrid, Spain; ^40^Department of Neuroscience, Canadian Centre for Behavioural Neuroscience, University of Lethbridge, Lethbridge, AB, Canada; ^41^Zilkha Neurogenetic Institute, Keck School of Medicine, University of Southern California, Los Angeles, CA, United States; ^42^Department of Pediatrics, University of California, Irvine, Irvine, CA, United States; ^43^The Russell H. Morgan Department of Radiology and Radiological Science, Johns Hopkins University School of Medicine, Baltimore, MD, United States; ^44^Institut d’Investigacions Biomèdiques August Pi i Sunyer (IDIBAPS), Àrea de Neurociències, Barcelona, Spain; ^45^Department d’Isquèmia Cerebral I Neurodegeneració, Institut d’Investigacions Biomèdiques de Barcelona (IIBB), Consejo Superior de Investigaciones Científicas (CSIC), Barcelona, Spain; ^46^Institute for Stroke and Dementia Research (ISD), Munich University Hospital, Munich, Germany; ^47^Graduate School of Systemic Neurosciences (GSN), Munich University Hospital, Munich, Germany; ^48^Munich Cluster of Systems Neurology (Synergy), Munich, Germany; ^49^Faculty of Medicine and University Hospital, Department of Neurology, University of Cologne, Cologne, Germany; ^50^Department of Neurology, Yale University School of Medicine, New Haven, CT, United States; ^51^Women’s Health in Neuroscience Program, Neuroscience and Experimental Therapeutics, Texas A&M College of Medicine, Bryan, TX, United States; ^52^Nuffield Department of Clinical Neurosciences, Wellcome Centre for Integrative Neuroimaging, University of Oxford, Oxford, United Kingdom; ^53^MRC Brain Network Dynamics Unit, University of Oxford, Oxford, United Kingdom; ^54^Department of Neurology, Pittsburgh Institute of Brain Disorders and Recovery, University of Pittsburgh, Pittsburgh, PA, United States; ^55^Department of Neurology and Neurotherapeutics, Peter O’Donnell Jr. Brain Institute, UT Southwestern Medical Center, Dallas, TX, United States; ^56^Pittsburgh Institute for Neurodegenerative Disorders, University of Pittsburgh, PA, United States; ^57^Department of Regenerative Medicine Research, Institute of Biomedical Research and Innovation, Kobe, Japan; ^58^Institute of Physics for Medicine Paris, INSERM U1273, ESPCI Paris, CNRS FRE 2031, PSL University, Paris, France; ^59^Department of Neurological Surgery, University of Wisconsin, Madison, WI, United States; ^60^UNICAEN, INSERM, INSERM UMR-S U1237, Physiopathology and Imaging for Neurological Disorders (PhIND), Normandy University, Caen, France; ^61^CHU Caen, Clinical Research Department, CHU de Caen Côte de Nacre, Caen, France; ^62^Department of Human Anatomy, College of Medical Sciences, Zhengzhou University, Zhengzhou, China; ^63^Department of Neurosurgery, Henry Ford Hospital, Detroit, MI, United States

**Keywords:** cell therapies, dementia, experimental therapy, hemorrhage, neuroprotection, neurorehabilitation, stroke, translational research

## Abstract

The past decade has brought tremendous progress in diagnostic and therapeutic options for cerebrovascular diseases as exemplified by the advent of thrombectomy in ischemic stroke, benefitting a steeply increasing number of stroke patients and potentially paving the way for a renaissance of neuroprotectants. Progress in basic science has been equally impressive. Based on a deeper understanding of pathomechanisms underlying cerebrovascular diseases, new therapeutic targets have been identified and novel treatment strategies such as pre- and post-conditioning methods were developed. Moreover, translationally relevant aspects are increasingly recognized in basic science studies, which is believed to increase their predictive value and the relevance of obtained findings for clinical application.This review reports key results from some of the most remarkable and encouraging achievements in neurovascular research that have been reported at the 10th International Symposium on Neuroprotection and Neurorepair. Basic science topics discussed herein focus on aspects such as neuroinflammation, extracellular vesicles, and the role of sex and age on stroke recovery. Translational reports highlighted endovascular techniques and targeted delivery methods, neurorehabilitation, advanced functional testing approaches for experimental studies, pre-and post-conditioning approaches as well as novel imaging and treatment strategies. Beyond ischemic stroke, particular emphasis was given on activities in the fields of traumatic brain injury and cerebral hemorrhage in which promising preclinical and clinical results have been reported. Although the number of neutral outcomes in clinical trials is still remarkably high when targeting cerebrovascular diseases, we begin to evidence stepwise but continuous progress towards novel treatment options. Advances in preclinical and translational research as reported herein are believed to have formed a solid foundation for this progress.

## Introduction

Overcoming more than two decades of translational standstill and providing options to treat selected stroke patients in extended time windows, the advent of recanalization approaches has sparked a new dynamic in preclinical and clinical stroke research. With a catheter in place, i.e., in a reopened cerebral artery, we now have the opportunity to apply neuroprotective treatments exactly when and where they may exert their optimal benefit (Savitz et al., 2019). These new options have the potential to initiate a “renaissance of neuroprotectants.” Neuroprotectants may also play an increasingly important role in the treatment of cerebral hemorrhages. Minimally invasive techniques for hematoma evacuation relying on specialized, catheter-based intervention strategies have been developed and were assessed in first clinical trials (Hanley et al., 2019). Neuroprotective agents may be delivered *via* those catheters once the hematoma was removed, and novel targets for neuroprotective interventions have been identified (Zille et al., 2017).

Nevertheless, the majority of patients still cannot benefit from the recent advances in acute interventions for cerebrovascular diseases. Among others, reasons comprise strict inclusion/exclusion and eligibility criteria, uncertainties regarding the optimal way to deliver such treatments as well as logistical challenges and limited availability of required infrastructure. Therefore, the need for new treatments being effective within therapeutic time windows beyond the range of hours as well as supporting long-term recovery and recuperation continues to exist. The development of these new treatments should take into account advances in basic science areas such as neuroimmunology, neurobiochemistry, and molecular biology, as well as cell-based approaches. Moreover, translational research shall reflect advances in imaging technologies and rehabilitation research and must consider aspects such as the influence of sex and age on therapeutic outcome.

The International Symposium on Neuroprotection and Neurorepair (ISN&N) discussed these aspects in the light of current therapeutic advances in cerebrovascular diseases. The ISN&N is a biennial meeting series on fundamental and translational neuroscience with a scope on cerebrovascular diseases and dementia (Boltze et al., 2011, 2012; Demuth et al., 2017). Attracting leading neuroscientists and clinicians from around the world, the meeting is characterized by intensive scientific exchange and discussions of new insights into neurovascular and neurodegenerative diseases. To share some of the most exciting reports with a wider academic community in a concise format, this review summarizes cross-selected, outstanding sessions from the 10th ISN&N that took place from October 9th to 11th 2018 in Radebeul near Dresden, Germany. It covers the main topics of the meeting, and puts a particular scope on translational and clinical aspects, as such providing a good overview of the field.

### Conditioning Medicine: Basic Mechanisms and Clinical Potential

It is now well accepted that ischemic pre- and post-conditioning promotes ischemic neuroprotection in different modalities (e.g., pharmacological, remote, and ischemic) and in many different animal models (Wang et al., 2015). Novel mechanisms involved in conditioning were discussed and clinical views of how to apply conditioning strategies to thrombectomy were provided.

IL (Madrid, Spain) presented that toll-like receptor 4 (TLR4) has both protective (ischemic tolerance) and damaging (acute ischemia) roles in cerebral ischemia, including hemorrhagic transformation (Pradillo et al., 2009; Vartanian et al., 2011; García-Culebras et al., 2017). TLR4 deficiency increased the levels of alternative neutrophils (N2), which may be involved in the resolution of inflammation and in the neuroprotective effect observed after stroke (García-Culebras et al., 2019). New drugs blocking TLR4 (Fernández et al., 2018) may be useful for the treatment of patients with stroke.

RAS (Pittsburgh, PA, USA) demonstrated that ischemic neuroprotection can be achieved by dietary restriction, improving both gray and white matter outcomes and functional recovery (Zhang et al., 2018). Although the activation of sirtuins may be a potential contributor to ischemic neuroprotection (Haigis and Guarente, 2006; Guarente, 2008; Stetler et al., 2014), dietary restriction is also well known to affect the release of a variety of humoral factors, particularly from adipose tissue. Adiponectin release into the plasma occurs acutely after dietary restriction and increased levels in the serum. This was associated with beneficial outcomes, as global knockout of adiponectin negated the effects of dietary restriction on the subsequent ischemic injury. The effects may be due to penetration of adiponectin into the brain parenchyma and the subsequent binding to its receptors on neural cells in the context of the opening of the blood-brain barrier (BBB) after ischemic injury (Brochu-Gaudreau et al., 2010; Zhang et al., 2018). Whether this scenario or the alternative situation—wherein adiponectin acts directly on endothelial barrier function to exert protection—underlies the neuroprotective effects remains under investigation.

MP-P (Miami, FL, USA) reported a novel window for ischemic tolerance for preconditioning mimetics, which was previously limited to 48–72 h after cerebral ischemia (Narayanan et al., 2015). Resveratrol preconditioning yielded gene expression changes in 136 genes at 14 days after single administration: many of the 116 downregulated genes were related to transcription, synaptic signaling, and neurotransmission, resembling the metabolic depression observed in hibernating species. This downregulation of gene expression fits well with the hippocampal synaptic and electrophysiological depression observed with pre-conditioning mimetics (DeFazio et al., 2009; Neumann et al., 2015; Cohan et al., 2017). Also, glycolysis and mitochondrial respiration efficiency were increased upon resveratrol preconditioning (Koronowski et al., 2017; Khoury et al., 2019), indicating an increased reliance on energy-producing pathways.

PL (Shanghai, China) discussed anesthetic postconditioning as a valuable neuroprotection candidate (Cohan et al., 2017) as fast onset and short-acting anesthetics allow smooth anesthesia induction and faster anesthetic emergence to overcome the clinical limitation that neurological assessment during anesthesia or sedation is not possible. New technology-based anesthetic drug delivery systems, such as target-controlled infusion, close loop infusion, and computer-assisted sedation systems allow better control of anesthesia (Struys et al., 2016; Zaouter et al., 2016). Dr. Li further reported about a planned clinical study in stroke patients undergoing endovascular thrombectomy to compare general anesthesia with and without anesthetic postconditioning for countering reperfusion injury after endovascular thrombectomy.

Overall, conditioning represents a clinically available therapeutic strategy for ischemic stroke, and the presented findings put forward our understanding of the underlying mechanisms and provided novel insights into its clinical application. However, to utilize its full potential, relevant aspects of heterogeneous patient populations such as age and sex have to be considered. Their relevance has already been shown in numerous studies.

### Effect of Sex and Age on Post-ischemic Outcome and Therapy

Sex and age are known to affect stroke outcomes (Kim and Vemuganti, 2015). Their contribution concerning mechanisms of ionic imbalance, inflammation, microRNAs, and the interaction of long noncoding RNAs with epigenetics was discussed.

As the expression of the chloride influx transporter, Na-K-Cl cotransporter 1 (NKCC1) in male rodent brains starts earlier during development compared to female brains, targeting NKCC1 during post-injury in adulthood may be sex-dependent. Indeed, DS (Pittsburgh, PA, USA) showed that pharmacological blockade of NKCC1 protein has higher sensitivity in adult male mice than female mice for reducing post-ischemic brain injury (Huang et al., 2019).

FL (Houston, TX, USA) presented that post-stroke inflammation and microglial activation are sex- and age-dependent and that these differences exist throughout the lifespan (Zhao et al., 2017b; Ritzel et al., 2018). He also showed that sex-chromosomal effects in addition to sex hormones contribute to the differential outcomes between males and females after focal ischemia (McCullough et al., 2016).

AR (Miami, FL, USA) reported that the depletion of endogenous estrogen at reproductive senescence increases inflammasome proteins in the brain of females, but not in age-matched male rats (de Rivero Vaccari et al., 2016). In reproductively senescent females, extracellular vesicles (EVs) from reproductive organs carry inflammasome proteins to the brain leading to an exacerbated innate immune response that may be responsible for the increased severity of ischemic damage in senescent females (D’Adesky et al., 2018).

FS (Bryan, TX, USA) showed that stroke neuroprotectants may act in a sex-specific manner. Post-stroke administration of IGF-1 is neuroprotective after stroke. Preventing microRNA let-7f, thus upregulating IGF-1 which is reduced in aging animals and humans, is protective in young female rats. However, it is not neuroprotective in young males and even worsens outcomes in senescent females post-stroke (Selvamani et al., 2012; Sohrabji et al., 2019). Let7f also regulates caspase 3, and treatment with miR-363–3p mimic (repressing caspase-3) protects young and senescent females, but not males, after focal ischemia (Selvamani and Sohrabji, 2017). Thus, the neurotoxic effect seen in older females may be related to an increase in caspase-3 and its cell death pathways caused by the blocking of let7f (Sohrabji and Selvamani, 2019). These findings also illustrate the complexity of sex-specific neuroprotective actions that must be taken into consideration.

RV (Madison, WI, USA) presented that the long noncoding RNAs Fos downstream transcript (FosDT) is induced after stroke and that knockdown of FosDT with a siRNA cocktail protects the brain in both males and females and also aged animals (Mehta et al., 2015). The epigenetic modification hydroxymethylation that converts 5-methylcytosine to 5-hydroxymethylcytosine induced after stroke is neuroprotective in both sexes (Morris-Blanco et al., 2019).

In summary, the contribution of age and sex to the outcome of cerebrovascular diseases should be carefully considered in the development of novel therapeutic strategies.

### Long-Term Neuroinflammation During Functional Recovery From Stroke

Inflammation has been regarded as a target to reduce brain damage in acute stroke. While dampening innate immune responses and addressing complement or danger signaling effectively reduces acute brain damage (Muhammad et al., 2008; Alawieh et al., 2018), certain inflammatory responses are necessary for repair as they induce cell migration, proliferation, matrix deposition, and tissue remodeling. This suggests that initial inflammatory reactions trigger a set of responses that may improve functional outcome in the long term.

AMP (Barcelona, Spain) reported that although monocytes and macrophages reach the brain within the first 4 days after stroke (Miró-Mur et al., 2016), macrophages persist for weeks in the damaged brain tissue (Wattananit et al., 2016; Mantovani, 2017). While they may have detrimental functions acutely, macrophages also play critical roles in long-term functional recovery (Wattananit et al., 2016). Besides, lymphocytes, which promote acute thromboinflammation through mechanisms that do not involve adaptive immune responses (Kleinschnitz et al., 2010), also infiltrate the brain in higher numbers during chronic phases, when adaptive immune responses may play a role in functional impairment (Selvaraj and Stowe, 2017).

AMS (Lexington, KY, USA) discussed that CD8^+^ T cells (i.e., cytotoxic T cells) move into several regions interconnected to the infarct within 4 days after transient middle cerebral artery occlusion (tMCAO) in mice, where they remain and support recovery (Poinsatte et al., 2019). Antibody-mediated depletion of CD8^+^ T cells improved motor recovery and reduced diapedesis of other leukocyte populations, including macrophages, neutrophils, and CD4^+^ T cells (Selvaraj and Stowe, 2017).

MSB (Stanford, CA, USA) described that in a model of post-stroke cognitive impairment, mice have normal cognition and hippocampal long-term potentiation 1 week after a cortical stroke adjacent to the hippocampus, while within 7 weeks, cognitive impairment and progressive loss of long-term potentiation begin (Doyle et al., 2015). In parallel, antibody-producing B-lymphocytes accumulate in the infarct core. Antibody levels increase in both the core and the surrounding brain parenchyma but the specificity of these antibodies could not (yet) be revealed. However, ablating B-lymphocytes prevents delayed cognitive impairment. Dr. Buckwalter further presented that B-lymphocytes are involved in this process in humans, suggesting that B cells may be critical targets for the development of post-stroke immunotherapies.

FS (Bryan, TX, USA) showed that anti-inflammatory miRNA therapy improves long-term affective dysfunction after ischemic stroke (Panta et al., 2019). Intravenous injection of mir363-3p mimics reduced infarct volume in the acute phase of stroke, ameliorated sensory-motor impairment, and attenuated transient increases of IL-6 and TNF-α. Depressive behaviors, assessed over 30–100 days after stroke, were also attenuated in mir363-3p treated animals (Panta et al., 2019). This indicates that a neuroprotectant that has anti-inflammatory properties may also improve affective dysfunction after stroke.

Taken together, these studies highlight the complex role of inflammation in the chronic phase after stroke and that modulating inflammation may require targeting a specific subset of inflammatory cells and proteins.

### Targeting the Interplay Between Stem Cells and Neuroinflammation in Stroke Regeneration

Sustained neuroinflammation is also required to eliminate degenerating tissue, supplying a restorative milieu for stem cell-mediated repair. However, immune responses are often dysregulated and lead to detrimental effects on regeneration. Recent work aims to elucidate the complex and interwoven processes of neuroinflammation and stem cell-mediated regeneration after a stroke that may form the basis for novel therapeutic approaches to modulate these processes and their interactions.

SUV (Cologne, Germany) reported that differentially polarized microglia differ in their effects on the fate of neural stem cells (NSC) *in vitro* and *in vivo*, with pro-inflammatory microglia promoting the generation of astrocytes, while anti-inflammatory microglia support neurogenesis. Regardless of their polarization, the secretome of activated microglia inhibits NSC proliferation, increases NSC migration, and accelerates NSC differentiation (Vay et al., 2018). These combined effects suggest that activated microglia induce NSC to exit the cell cycle, to migrate towards a putative lesion site, and to attempt cell replacement by differentiation. Besides, NSC promotes functional restoration after stroke not only by cell replacement but also by modulating surrounding cells and inflammatory processes.

ZK (Lund, Sweden) discussed the significance of monocyte-derived macrophages (MDM) in supporting regenerative processes after ischemic stroke. MDM are recruited to the ischemic lesion site exhibiting a high degree of functional plasticity, changing from a pro- to an anti-inflammatory phenotype during the first weeks after stroke. Blockage of MDM recruitment worsens functional and structural outcomes after stroke (Wattananit et al., 2016; Kronenberg et al., 2018). Dr. Kokaia’s work suggests that immune cells affect the regenerative capacity of the brain, most likely by modulating stem-cell-derived neuronal plasticity in the subventricular zone and the adjacent striatum (Laterza et al., 2017).

KG (Berlin, Germany) emphasized the beneficial role of anti-inflammatory MDM activated in the early phase after stroke. However, not only stroke-invading MDM but also brain-resident microglia are activated, and both MDM and microglia often display a mixture of phenotypic markers (“intermediate states”) during the first weeks after stroke (Kronenberg et al., 2018).

Overall, the cross-talk between inflammatory cells acting as key regulators and endogenous or grafted neural stem cells acting as key effectors is highly relevant during post-stroke regeneration. Thus, therapeutic strategies based on modulating these cell entities and their interactions could be exploited to facilitate functional recovery after stroke.

### Extracellular Vesicles: Therapy and Imaging

Although there is growing evidence that long-term benefits are produced by stem cells, particularly in sudden onset diseases, these cells are often short-lived after transplantation (Kim et al., 2012; Jablonska et al., 2016). Stem cells release EVs including exosomes that mediate therapeutic effects (Xin et al., 2012, 2013a; Zhang et al., 2015) and in certain conditions, they can achieve the same efficacy obtained by the administration of the stem cells themselves (Doeppner et al., 2015).

TD (Göttingen, Germany) explained the positive effects of mesenchymal stem cell-derived EVs (Doeppner et al., 2015). Interestingly, similar effects have recently been described for EVs derived from neural progenitor cells (Zheng et al., 2021). This suggests that EV-derived modes of action could be a general mechanism related to beneficial stem and progenitor cell effects. Apart from this, Dr. Doeppner also reported innovative therapeutic approaches such as the application of remote ischemic post-conditioning (for review and current clinical application please see Ji et al., 2019), defined as a transient and subcritical period of peripheral organ ischemia following cerebral ischemia, to exert beneficial but transient effects on recovery. Interestingly, post-conditioning also facilitated intracerebral transplantation of neural progenitor cells resulting in persistent protection and improved functional recovery (Doeppner et al., 2017).

YX (Detroit, MI, USA) discussed that exosomes elicit potent cellular responses *in vitro* and *in vivo* by delivering their cargos, including lipids, proteins, RNAs (miRNAs and mRNAs), and other macromolecules, to recipient cells (Xin et al., 2013a; Zhang et al., 2019). In turn, microRNAs regulate numerous genes that mediate beneficial therapeutic effects (Zhang et al., 2019) and exosomes with select miRNAs can be designed to provide further improved therapeutic efficacy (Xin et al., 2013b, 2017).

MJ (Baltimore, MD, USA) explained strategies for EV labeling and imaging to assess direct routes of EV delivery that are potentially more beneficial and to monitor EV biodistribution in the body. Iron oxide-based labeling is comparably easy as it is sufficient to apply a standard labeling protocol to stem cells, from which EVs are subsequently derived (Busato et al., 2017; Dabrowska et al., 2018). While the signal is strong, its specificity is quite low due to other sources of hypointensities in tissues related to pathology. However, this approach may be particularly compelling to monitor EV delivery in real-time as the signal of the label can be subtracted from the background image obtained before the procedure (Walczak et al., 2017). Further labeling techniques based on radioisotopes are very attractive to assess EV biodistribution even in clinical settings, while other modalities such as fluorescence or bioluminescence labeling are limited to preclinical studies (Choi and Lee, 2016).

Summing up, cell-free exosome-based approaches may replace stem cells and become a major driving force for therapeutic interventions with the aid of engineering and imaging.

### Traumatic Brain Injury Can Lead to Cerebrovascular Disease

A provocative question discussed was whether traumatic brain injury can serve as a model to study neurovascular unit dysfunction in cerebrovascular disease as their relationship has been proposed in recent studies (Pop and Badaut, 2011; Sandsmark et al., 2019).

NP (Munich, Germany) showed that controlled cortical impact in adult mice induces progressive cognitive decline (Mao et al., 2020). While motor function recovered partially after TBI, depression-like behavior and the loss of memory function were progressive. Increasing tissue loss in the corpus callosum, hippocampus, and hydrocephalus formation was also observed over time. Hence, experimental TBI in mice replicates the long-term sequelae post-trauma including dementia and depression that are observed in humans. It needs to be determined whether these changes are triggered by concomitant vascular injury or other, so far unknown mechanisms.

JB (Bordeaux, France) discussed the alteration of the NVU months after mild pediatric TBI in which changes of water channel expression on astrocytic endfeet adjacent to blood vessels have been observed (Fukuda et al., 2013). These changes are related to diffusion tensor imaging modifications in different brain structures at 12 months post-injury after a single impact.

Linked with NVU remodeling, AO (Irvine, CA, USA) presented the importance of the activation of the Wnt-Beta-catenin pathway in cerebral blood vessels after TBI (Salehi et al., 2018). After acute vascular loss post-TBI, new immature vessels occurred at the injury site 7 days after the injury. This coincided with decreased levels of β-catenin protein in the injury site but dramatically increased β-catenin protein expression in vessels. β-catenin and Wnt expression were particularly increased in perilesional vessels and are thought to promote new vessel formation into the injury site.

Overall, most of the pre-clinical models of TBI from mild to severe severities reproduce the long-term clinical changes described in the literature in terms of neuroimaging. IKK (Boston, MA, USA) reviewed noninvasive neuroimaging techniques that can help to bridge clinical and preclinical studies.

In summary, the presented data impressively showed that assessing long-term outcomes after TBI in preclinical models may be a suitable approach for a better understanding of the molecular and cellular processes in cerebrovascular diseases.

### Targeting Acute Pathomechanisms Following Subarachnoid Hemorrhage

Subarachnoid hemorrhage (SAH) is a devastating type of stroke with a high mortality rate, especially during the acute phase after the ictus. Therefore, current strategies focus on minimizing early brain injury to reduce SAH-induced mortality and disability (Lantigua et al., 2015).

NP (Munich, Germany) reported that SAH causes acute and long-lasting constrictions of pial arterioles (microvascular spasm): the CO_2_-reactivity is lost within the first 3 h, followed by an impairment of neurovascular coupling within 24 h after SAH. Within 1 month, CO_2_-reactivity reestablishes, while neurovascular coupling does not recover (Balbi et al., 2017, 2020). Loss of neurovascular coupling may result in further brain damage after SAH due to an uncoupling of blood flow and metabolism, e.g., during cortical spreading depolarizations or even during normal brain activity.

SAH also induces acute spreading depolarizations as shown by JPD (Berlin, Germany) both in animal models and in patients (Dreier et al., 2018). These lead to severe hypoperfusion in tissue at risk for progressive injury, which may directly initiate infarction (Lückl et al., 2018).

RK (Ann Arbor, MI, USA) demonstrated that SAH induces BBB disruption (Egashira et al., 2016) and white matter injury acutely after the ictus (Guo et al., 2017). Dr. Keep further discussed the importance of clot-derived factors and the significance of hemorrhage extension into the ventricular system leading to injury and hydrocephalus. In particular, epiplexus cell activation was associated with hydrocephalus development and may occur in response to thrombin production after SAH (Wan et al., 2019).

BAG (Newcastle, Newcastle upon Tyne, UK) presented the ongoing Treatment of Poor-grade SubArachnoid Hemorrhage Trial 2 (TOPSAT2; ISRCTN15960635) that investigates whether early treatment compared to conventional treatment after neurological recovery improves functional outcome after 12 months in poor-grade aneurysmal SAH patients.

In summary, these reports demonstrated several acute pathomechanisms after SAH that present promising therapeutic targets that need to be further assessed in clinical trials of SAH.

### Targeting the Hematoma and the Search for Protective Strategies in Intracerebral Hemorrhage

Another hemorrhagic subtype of stroke is intracerebral hemorrhage (ICH), which remains a major cause of mortality and permanent disability (Poon et al., 2014; van Asch et al., 2010). Recent studies targeting both primary and secondary injury components (Keep et al., 2012) were discussed in two mini-symposia of the 10th ISN&N.

As targeting primary injury by surgical evacuation using craniotomy does not improve functional outcome in large surgical trials of ICH (STICH I and II; Potts and Riina, 2014), recent efforts focused on Minimally Invasive Surgery with Thrombolysis in ICH Evacuation (MISTIE). MISTIE featured the administration of alteplase (initially 1.0 mg in ml followed by up to nine 3 ml flushes every 8 h) *via* a soft catheter directly into a residual hematoma that was primarily addressed by clot aspiration (Hanley et al., 2019). DFH (Baltimore, MD, USA) reported no overall efficacy of the MISTIE procedure in phase III clinical trial. However, a subgroup analysis on the surgical performance showed that a hematoma reduction below 15 ml increases the chances of having a good outcome by 10% for each additional ml of hematoma removed (Awad et al., 2019). Furthermore, KRD (Miami, FL, USA) demonstrated that a novel hemostatic agent, red blood cell-derived microparticles (RMPs), which display negatively charged phosphatidylserines to induce coagulation, limited hematoma growth in a collagenase-induced model of ICH in rats. According to the pharmacokinetic and safety profile of RMPs, this agent is applicable in the clinics (Jy et al., 2018; Rehni et al., 2019). Since a complete evacuation of the hematoma is unlikely to be achieved in every patient, additional strategies targeting clot-derived factors are needed for ICH therapy.

Addressing neuroinflammation as a secondary injury component, LHS (Yale, New Haven, CT, USA) described the properties of leukocytes in the peripheral blood and hematoma evacuates from the MISTIE III clinical trial using RNA sequencing and provided evidence for the importance of efferocytosis in reducing inflammation in human macrophages (Chang et al., 2018). Furthermore, macrophages and microglia undergo acute activation during the first week after ICH, with microglia producing inflammatory cytokines and chemokines, and a decreasing expression of microglia-specific genes (Taylor et al., 2017). Other immune cells such as neutrophils are another source of damage and secondary injury as they infiltrate the brain in response to sterile inflammation triggered by ischemic stroke or ICH. JA (Houston, TX, USA) presented that neutrophils are plastic regarding their phenotype and the cytokine IL-27 can alter their gene expression during maturation in the bone marrow (Zhao et al., 2017a). After ICH, IL-27-modified neutrophils can deliver to the hematoma site more beneficial haptoglobin and lactoferrin to neutralize the toxicity of hemoglobin and iron and to limit the brain exposure to pro-oxidative and pro-inflammatory mediators normally transported by the infiltrating neutrophils.

As hemolysis leads to the release of blood breakdown products that induce cell death, targeting cell death is another strategy for secondary intervention. MZ (Lübeck, Germany) demonstrated that primary cortical neurons in culture die by a mixture of ferroptotic and necroptotic cell death when exposed to blood breakdown products (Zille et al., 2017). In contrast, cell death mechanisms underlying brain endothelial cell death after hemorrhagic stroke remain poorly understood (Zille et al., 2019). JW (Baltimore, MD, USA) demonstrated the co-existence of ultrastructural characteristics of ferroptosis, autophagy, and necroptosis after experimental ICH *in vivo* (Li et al., 2018). The inhibition of ferroptosis using the small molecule ferrostatin-1 improved neurological function and conferred protection after ICH (Li et al., 2017). Moreover, SSK (White Plains, NY, USA) showed that the ferroptosis inhibitor N-acetylcysteine improved functional recovery after ICH in mice by inhibiting toxic lipid products (Karuppagounder et al., 2018).

**Figure 1:**
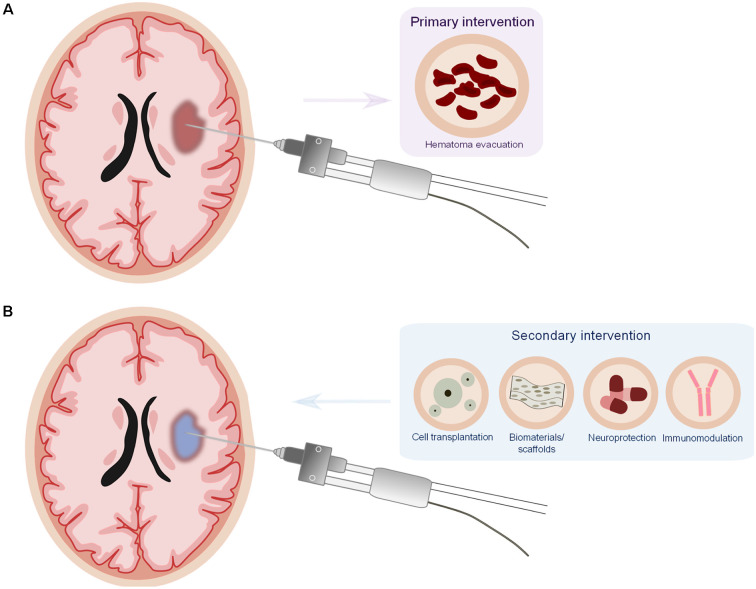
Proposed model for a combinatorial therapy in intracerebral hemorrhage (ICH). **(A)** Minimally invasive surgery for the evacuation of the hematoma (primaryintervention). **(B)** Minimally invasive surgery offers the particular opportunity to deliver potential therapeutics (cell transplantation, biomaterials/scaffolds, neuroprotective, and/or immunomodulatorydrugs) as secondary intervention directly into the lesion site, reducing drug delivery issues and potential systemic side effects of the drugs.

Taken together, the presented studies put forward the idea that targeting the hematoma is an important strategy in ICH therapy and that there should be a combinatorial approach consisting of primary intervention (to evacuate the hematoma) together with a secondary intervention (immunomodulatory and neuroprotective; Figure 1).

### Next-Generation Functional Testing in Experimental Brain Research

The reliable assessment of functional improvement after cerebrovascular injury and potential treatment remains challenging. The recent development of new assessment strategies in experimental brain research considers several conceptual and methodological aspects.

GASM (Lethbridge, AB, Canada) showed that compensation after brain injury represents the primary process of functional improvement, i.e., the development of alternate behavioral strategies rather than the recovery of the original functions (Metz et al., 2005; Fouad et al., 2013). Compensatory behaviors are clearly recognizable and acquired through repetitive training in a task-specific manner (Girgis et al., 2007; Kirkland et al., 2012).

KF (Edmonton, AB, Canada) discussed recent advances that have exploited automated test apparatus solutions to enhance the efficacy of animal training and assessment (Fenrich et al., 2015; Bova et al., 2019) along with machine learning algorithms to identify discrete aspects of compensatory movement patterns (Ryait et al., 2019).

EH (Montreal, QC, Canada) showed that exercise-based rehabilitation and pharmacotherapy influence white matter inflammation and the degree of cognitive and cerebrovascular improvement, including neurovascular coupling, in a mouse model of vascular cognitive impairment and dementia (Trigiani and Hamel, 2017; Trigiani et al., 2019).

These observations open new avenues for the discovery of biomarkers that aid in the stratification of patients to personalized rehabilitation programs. The overarching goal of combined multi-level functional and molecular analysis is the improved translation of experimental innovations to clinical practice.

### Cutting-Edge Brain Imaging in Disease and Regeneration

Magnetic resonance imaging (MRI) has been decisive in understanding cerebrovascular disease over the past decades. Besides MRI, functional ultrasound has recently evolved as an analog to functional MRI. Cutting-edge imaging-based findings were reported at the 10th ISN&N.

AM (Los Angeles, CA, USA) reported on brain imaging of vascular dysfunction to understand BBB breakdown and how this may contribute to cerebrovascular diseases, with a particular focus on dementia. Measuring BBB alterations *in vivo* can be challenging. Examples of how brain imaging (i.e., dynamic contrast-enhanced MRI) may contribute to a better diagnosis and prognosis of BBB alterations and how targeting the BBB can influence the course of neurological disorders have been discussed. Finally, an outlook on future approaches to better understand the cellular and molecular events underlying mechanisms between BBB breakdown and neurodegeneration was given (Montagne et al., 2017).

DV (Caen Cedex, France) focused on molecular MRI of neuroinflammation, including targeting vascular cell adhesion molecule 1 and P-selectin. The perspectives for the clinical application of this technology were discussed and he showed that molecular MRI of adhesion molecules, using micro-sized particles of iron oxide complexed with specific antibodies of adhesion molecules, may be useful for the diagnosis and prognosis of brain disorders (Gauberti et al., 2018).

RMD (Utrecht, The Netherlands) presented recent data that inform on correspondences between quantitative functional and structural connectivity measures in the mammalian brain from resting-state functional MRI, diffusion-weighted MRI, and neuronal tract tracing. Overall, despite methodological differences between studies, the current literature supports the hypothesis that structural connectivity provides the hardware from which functional connectivity emerges (Straathof et al., 2019). However, strong structural connections are not necessarily strong functional connections.

MT (Paris, France) spoke on recent progress in ultrafast ultrasound imaging. Neurologists and neuroradiologists warrant an imaging technique that can be performed at the bedside (as transcranial Doppler ultrasound), is repeatable (as arterial spin labeling MRI), provides quantitative measurement (as positron emission tomography), and measures multiple perfusion parameters (as perfusion computed tomography). The recent development of ultrafast ultrasound sequences and advances in high-frequency probes technologies combines all these criteria in a unique system. Specifically, it allows detecting subtle cerebral blood volume changes due to neurovascular coupling, which has led to the development of the ultrasound analog to functional MRI, functional ultrasound providing a high spatiotemporal resolution of cerebral blood flow and brain functioning in animals and humans (Rabut et al., 2019).

The presented imaging tools promise to further enhance our understanding of cerebrovascular disease in the future that will form the basis for the development of novel therapeutic strategies. Moreover, these new imaging techniques might develop into invaluable diagnostic tools augmenting current diagnostic procedures and leading to better clinical treatment by identifying patients with specific dysfunctions that would benefit from targeted interventions. They may also provide opportunities to use imaging biomarkers of vascular dysfunction in patients with TBI or cerebral small vessel disease.

### Endovascular Approaches to Image and Treat Stroke: Beyond Thrombectomy

Modern mechanical thrombectomy techniques are highly efficient and have revolutionized the treatment of acute stroke patients with large vessel occlusion. However, the restoration of blood flow is insufficient to completely reverse the ischemic insult. The next phase of stroke intervention should therefore focus on reparative mechanisms that can be delivered with image-guidance (Chu et al., 2018) in the same setting after thrombectomy to further improve patient outcomes, as outlined by MP (Baltimore, MD, USA).

**Figure 2:**
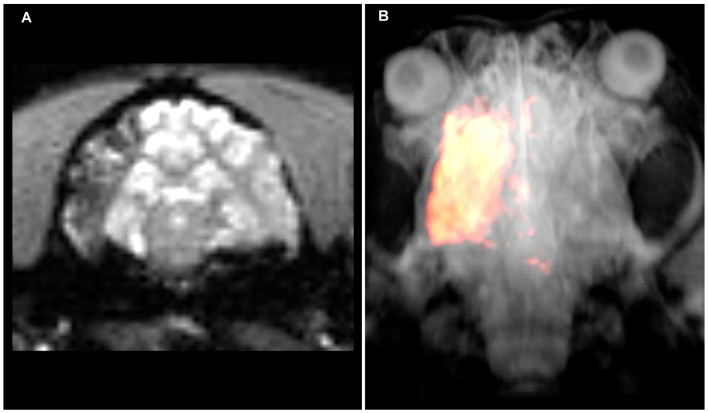
Intraarterial magnetic resonance imaging (MRI)-guided injection of mesenchymal stem cells into the canine brain. **(A)** Raw T2*w MRI after intraarterial injection of superparamagnetic iron oxide particle-labeled mesenchymal stem cells showing cell accumulation throughout the ipsilateral middle cerebral artery territory. **(B)** Segmentation of hypo-intense pixels and 3D visualization of stem cell biodistribution. Please see the Supplementary Video 1 for a dynamic visualization of stem cell injection.

PW (Baltimore, MD, USA) stressed that the brain remains to be a challenging target for drug delivery with systemic injection frequently resulting in less than 1% of injected dose reaching the brain parenchyma. Due to compromised perfusion, drug accumulation in the stroke lesion is related to even higher uncertainty and unpredictability. Dr. Walczak presented approaches for spatially selective, intra-arterial drug and cell delivery guided with interventional MRI (Figure 2; Supplementary Video 1). High precision and reproducibility are achieved by visualization of trans-catheter pre-injections of a contrast agent using dynamic MRI. Imaging the trans-catheter perfusion territory facilitates optimization of that territory by modulating the infusion rate or the position of the catheter tip. Once the targeted volume is optimized, the drug (or any other therapeutic agent) is infused. This procedure improves the precision and efficiency of targeting (Walczak et al., 2017). In case BBB opening is desired or required, it can be implemented in the procedure by an additional step such as infusion of hyperosmotic mannitol (Chu et al., 2018).

As a new tomographic imaging method, PL (Hamburg, Germany) reported on magnetic particle imaging, which is a useful tool for the rapid assessment of the cerebral vasculature and perfusion in stroke due to its temporal resolution. Magnetic particle imaging provides great innovation potential to improve stroke imaging and treatment in the future (Ludewig et al., 2017).

DG (Olsztyn, Poland) presented a recently developed endovascular method for inducing ischemic stroke in swine (Golubczyk et al., 2020). To overcome the long-standing drawback due to the vascular rete preventing the navigation of endovascular catheters into the brain, her approach is to inject thrombin solution *via* the pharyngeal ascendant artery to occlude cerebral vessels. The entire procedure including the injection of a clot-forming solution, the blockage of vasculature and the evolution of stroke was visualized with MRI. This new model offers several important advantages including a minimally invasive approach in a species with large, gyrencephalic brain and overall high clinical relevance (Herrmann et al., 2019).

A potential treatment option, as outlined above, is targeting neuroinflammation. MG (Hamburg, Germany) reported on the influence of the post-ischemic inflammatory reaction on the fate of the ischemic brain (Gülke et al., 2018). He presented novel data on conserved proinflammatory pathways, which are crucial for the initiation of the damaging inflammatory reaction in the ischemic brain. Finally, he correlated the available experimental data with human clinical studies.

Taken together, the described tools and strategies are particularly relevant at the time of growing use of endovascular mechanical thrombectomy as the placement of intra-arterial catheters introduces the opportunity to implement intra-arterial adjuvant therapies.

### Recent Advances in Neurorehabilitation Strategies in Stroke

As a result of improved acute stroke care, more and more patients survive, but many are left with permanent disabilities, making stroke a major cause of adult disability. Although neurorehabilitation is the only established stroke therapy being effective beyond acute stages of the disease, combinatorial approaches may strengthen the beneficial effects of neurorehabilitation strategies.

MC (Pisa, Italy) described rehabilitation of motor function in a mouse model of focal cortical stroke induced by photothrombosis. A robotic device (M-platform) was used for the daily training of the affected forelimb in combination with pharmacological contralesional silencing to dampen excessive transcallosal inhibition. Mice receiving this combined therapy displayed motor improvements that persisted beyond the treatment period and these were even generalized to non-trained tasks (Spalletti et al., 2017).

JJ (Kuopio, Finland) presented on combined cell therapy and rehabilitative training in experimental stroke. The transplantation of subventricular zone or human embryonic stem cells into the perilesional cortex was characterized by low cell survival (Hicks et al., 2009). Nonetheless, some improvement was observed in behavioral outcomes both when cells were transplanted alone or together with housing the experimental animals in an enriched environment i.e., mimicking rehabilitation. Likewise, the intravenous delivery of human adipose tissue-derived mesenchymal stem cells and exposure to the enriched environment improved forelimb function in the cylinder test and sticky label test (Mu et al., 2019).

CJS (Oxford, UK) discussed the role of non-invasive brain stimulation in recovery, which is a promising tool as it is relatively cheap, very well tolerated, and easy to use. However, the functional improvements demonstrated are highly variable across people. Various MRI approaches have been applied to predict the response to transcranial direct current stimulation in the motor system, indicating that a reduction of inhibition in the primary motor cortex (M1) is a central mechanism for motor plasticity in humans, both in health and in recovery after stroke (Stagg et al., 2011; Blicher et al., 2015; Kolasinski et al., 2019). Indeed, GABA levels in the ipsilateral M1 cortex can predict the response to transcranial direct current stimulation (O’Shea et al., 2014).

FCH (Geneva, Switzerland) summarized the reasons for heterogeneous results of neurorehabilitative interventions, such as non-invasive brain stimulation, and discussed a paradigm shift from imprecision of “one treatment suits all” strategies towards personalized medicine approaches delivered in a manner tailored to the demands of the individual patient (Raffin and Hummel, 2018; Coscia et al., 2019). Structural MRI imaging and connectome analyses can be used to determine the functional outcome of patients to stratify patients towards specific brain stimulation protocols (Koch and Hummel, 2017; Schulz et al., 2017).

Taken together, these studies support the use of rehabilitation and neuromodulatory approaches for promoting post-stroke recovery. Although combination therapy and early delivery may be more effective, further studies with greater statistical power to cope with these complex experimental designs will be needed to determine the optimal treatment protocol, the effect of co-morbidities, and to explore underlying therapeutic mechanisms (Boltze et al., 2017). Moreover, novel imaging approaches as discussed in the section “Cutting-Edge Brain Imaging in Disease and Regeneration” (above) might be helpful to identify patients that may benefit from brain stimulation interventions.

## Outlook and Conclusions

Based on the setbacks seen in the past decades of cerebrovascular disease research, the field has clearly consolidated and recognized the reasons for previous translational failure as exemplified in the field of cell therapies (Cui et al., 2019). Recent guidelines for translational research in that field stress, for instance, the importance of considering comorbidities, the use of highly predictive animal models and experimental settings as well as a closer interaction between preclinical and clinical research activities for more integrated study designs being able to inform each other (Boltze et al., 2019). Moreover, with the refinement of our research tools, some of which have been discussed herein, and increasing study quality (Ramirez et al., 2017), it seems that the translational roadblock is slowly overcome as new therapeutic concepts start to arise (Liebeskind et al., 2018). For instance, new combinations of recanalization and neuroprotecive approaches are believed to emerge for stroke (Savitz et al., 2019) and potentially ICH.

Encouraging progress is also witnessed in related fields such as vascular dementia or even Alzheimer’s disease. For instance, significant progress has been made in understanding the vascular contribution to various form of dementia (Iadecola and Gottesman, 2018; Royea et al., 2020). While the role of the frequent but treatable risk factors of dementia such as hypertension and hypercholesterinemia is better understood (Kaiser et al., 2014; Trigiani et al., 2019), new animal models of vascular cognitive impairment may facilitate translational research programs (Hainsworth et al., 2017). In parallel, new targets for pharmacological interventions have been identified (Loera-Valencia et al., 2019) and are expected to lead to future clinical trials (Howard et al., 2019). Although there will likely be setbacks on the way forward and we shall avoid premature enthusiasm as seen in the past, the “therapeutic nihilism” still observed in translational cerebrovascular research just 5 years ago has turned into new optimism.

The 11th ISN&N will continue to monitor the progress in the fields of basic translational and clinical cerebrovascular disease research. Moreover, it will extend its scope by partnering with the 18th International Conference on Brain Edema and Cellular Injury (BEM) for a single combined meeting in October 2022 in Berlin, Germany.

## Author Contributions

All authors listed have made a substantial, direct and intellectual contribution to the work, and approved it for publication.

## Conflict of Interest

The authors declare that the research was conducted in the absence of any commercial or financial relationships that could be construed as a potential conflict of interest. The reviewer AL declared a shared affiliation, though no other collaboration, with several of the authors (IK, NP) to the handling Editor.
